# Treatment with *Helicobacter pylori*-derived VacA attenuates allergic airway disease

**DOI:** 10.3389/fimmu.2023.1092801

**Published:** 2023-01-24

**Authors:** Sebastian Reuter, Jonas Raspe, Hendrik Uebner, Alexandros Contoyannis, Eva Pastille, Astrid M. Westendorf, Georgia C. Caso, Timothy L. Cover, Anne Müller, Christian Taube

**Affiliations:** ^1^ Department of Pulmonary Medicine, Experimental Pneumology, University Hospital Essen- Ruhrlandklinik, Essen, Germany; ^2^ Institute of Medical Microbiology, University Hospital Essen, University of Duisburg-Essen, Essen, Germany; ^3^ Department of Pathology, Microbiology, and Immunology, Vanderbilt University Medical Center, Nashville, TN, United States; ^4^ Veterans Affairs Tennessee Valley Healthcare System Nashville, Nashville, TN, United States; ^5^ Institute of Molecular Cancer Research, University of Zurich, Zurich, Switzerland

**Keywords:** asthma, helicobacter pylori, vacuolating cytotoxin A (VacA), therapy, regulatory T cells

## Abstract

**Background:**

Asthma is an incurable heterogeneous disease with variations in clinical and underlying immunological phenotype. New approaches could help to support existing therapy concepts. Neonatal infection of mice with *Helicobacter pylori* or administration of *H. pylori*-derived extracts or molecules after birth have been shown to prevent the development of allergic airway disease later in life. This study evaluated the potential therapeutic efficacy of *H. pylori* vacuolating cytotoxin A (VacA) in allergic airway inflammation and investigated the underlying immunological mechanisms for its actions.

**Methods:**

Murine models of allergic airway diseases, and murine and human *in vitro* models were used.

**Results:**

In both an acute model and a therapeutic house dust mite model of allergic airway disease, treatment with *H. pylori*-derived VacA reduced several asthma hallmarks, including airway hyperresponsiveness, inflammation and goblet cell metaplasia. Flow cytometry and ELISA analyses revealed induction of tolerogenic dendritic cells (DC) and FoxP3 positive regulatory T cells (Tregs), and a shift in the composition of allergen-specific immunoglobulins. Depletion of Tregs during treatment with VacA reversed treatment-mediated suppression of allergic airway disease. Human monocyte derived DCs (moDC) that were exposed to VacA induced Tregs in co-cultured naïve autologous T cells, replicating key observations made *in vivo*.

**Conclusion:**

*H. pylori*-derived VacA suppressed allergic airway inflammation *via* induction of Tregs in both allergic airway disease models. These data suggest that the immunomodulatory activity of VacA could potentially be exploited for the prevention and treatment of allergic airway disease.

## 1 Introduction

Asthma is one of the most common noncommunicable diseases, affecting an estimated 358.2 million people worldwide (1). There is now in-depth knowledge about the pathophysiology of asthma, and important underlying mechanisms have been identified (2). However, all currently available treatments (including biologics) must be given over the long term, and no cure for asthma is currently available.

Bacteria are known to interact with the host through symbiotic, commensal or pathogenic host-bacteria relationships and can exert immune-modulatory effects. There is mounting evidence that interactions between microbes and the immune system are an important factor influencing susceptibility to allergic disease and asthma (3). The gastric bacterium *Helicobacter pylori* has the ability to induce immune suppression that, on one hand prevents clearance of the bacteria and on the other hand diminishes the risk of allergic diseases (4).

Data from epidemiological studies (5–8) that has been corroborated by studies in murine models (3, 9) suggest a protective effect of *H. pylori* to reduce the incidence of allergic asthma, hay fever, and other allergic disease manifestations. In addition, animal data suggest that exposure of mothers to *H. pylori* during pregnancy can induce a tolerogenic immunophenotype in their pups that attenuates the development of allergic diseases later in life (10). In this setting, the effects of *H. pylori* on allergic airway disease were found to be mediated by the modulation of dendritic cell (DC) and T cell responses (3, 9). However, all currently available data come from postnatal prophylactic models in which infection or administration of bacterial lysates were performed long before allergen sensitization and/or challenge.

In these models, using *H. pylori* strains lacking specific bacterial proteins showed that vacuolating cytotoxin A (VacA) plays a central role in mediating the immunosuppressive effects of the *H. pylori* bacterial infection (3). *H. pylori*-derived VacA is a secreted bacterial immunomodulator that differs in sequence and structure from other known bacterial virulence and persistence factors (11). Best known for its pore-forming function, it plays an important role in *H. pylori* colonization of the stomach (11). VacA can interact with different structural and immune cells, thereby influencing cellular mechanisms in several ways (12).

This study investigated the potential effectiveness of VacA as a therapeutic intervention for the treatment of allergic airway disease. To achieve this, purified *H. pylori*-derived VacA and recombinant VacA (rVacA) were assessed in acute and therapeutic models of allergic airway disease. In addition, a murine Treg depletion model, and murine and human DC/T cell co-cultures were used to determine the mechanistic basis by which VacA modulates the severity of allergic asthma.

## 2 Material and methods

### 2.1 Animals

C57BL/6J mice [Janvier Labs] were housed in the Laboratory Animal Facility of the University Hospital Essen. All mice were females and used at the age of 8–12 weeks. C.B6-Tg(Foxp3-DTR/EGFP)23.2Spar/Mmjax (DEREG) mice were kindly provided by Astrid Westendorf (Institute of Medical Microbiology, University Hospital Essen, University of Duisburg-Essen, Essen). These mice express the diphtheria toxin (DT) receptor and green fluorescent protein (GFP) under control of the FOXP3 promoter. Application of DT results in depletion of Tregs (13). Male and female DEREG mice aged 8–15 weeks were evenly distributed across all experimental groups.

All animal procedures were conducted in accordance with current federal, state, and institutional guidelines, and all experiments were approved by local regulatory authorities (Landesamt für Natur, Umwelt und Verbraucherschutz North Rhine Westphalia, Germany; reference number: AZ 81-02.04.2018.A084).

### 2.2 HDM

To mimic a sensitization with a human relevant allergen, extract derived from whole bodies of Dermatophagoides pteronyssinus was used in the experiments. D. pteronyssinus is one of the most frequently found mite species in german households represents the major allergen source of atopic people with a sensitization towards house dust mite in Europe (14).

HDM was obtained from Greer Laboratories [# XPB82D3A2.5]. The delivered lyophilized cake was reconstituted with PBS to a concentration of 1mg/mL total protein containing 13µg/mL Der p 1.

### 2.3 Experimental protocol

#### 2.3.1 Acute HDM-specific murine allergic airway disease model

**Figure 1:**
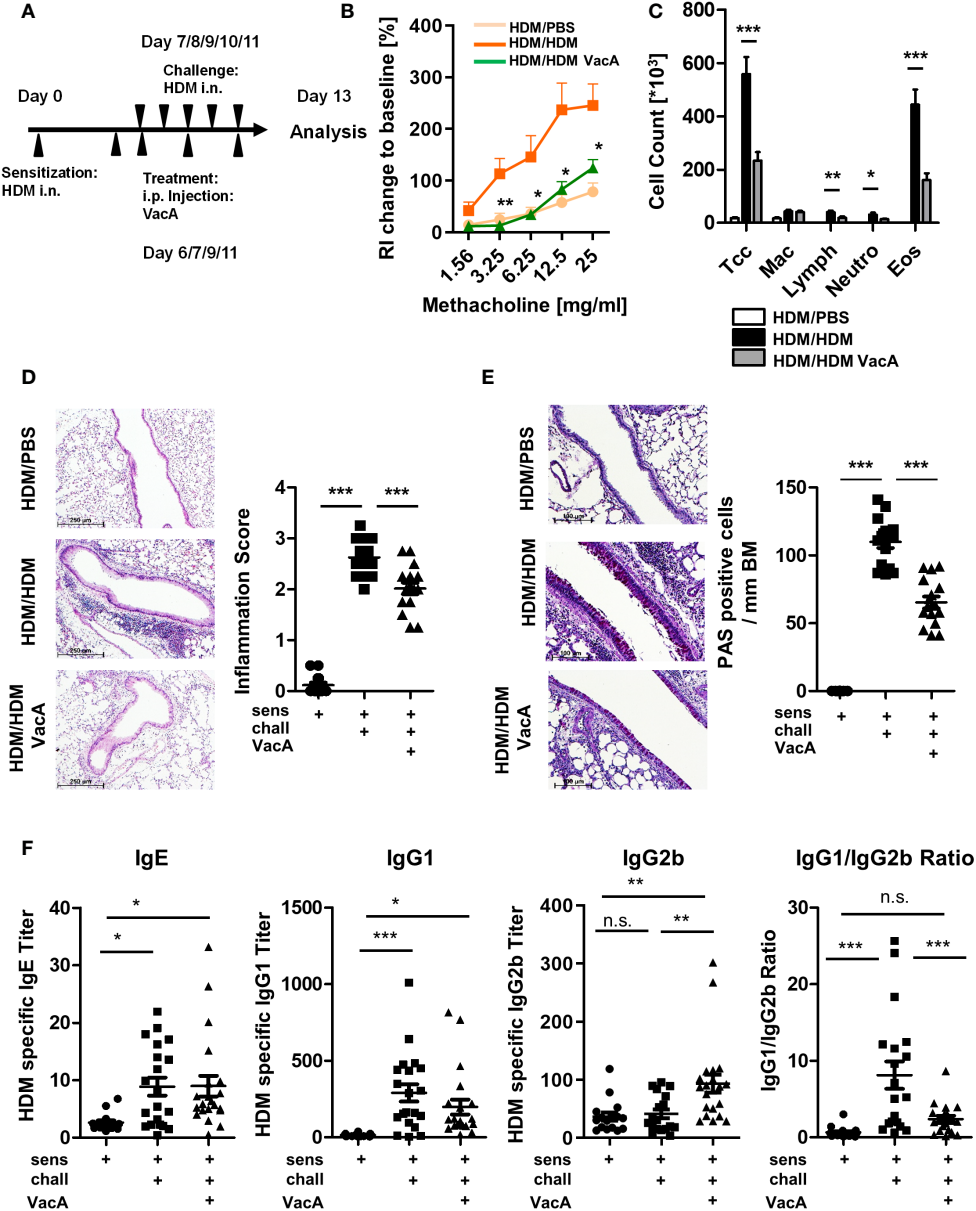
VacA treatment attenuates the asthma phenotype. **(A)** Animals were sensitized (day 0) and challenged intranasally (day 7–11) with house dust mite (positive control, HDM/HDM). VacA (HDM/HDM VacA) was given intraperitoneally (i.p.) on days 6, 7, 9 and 11. Phosphate-buffered saline (PBS)-challenged animals served as negative controls (HDM/PBS). **(B)** Change of airway resistance (RI): percentage change of airway resistance in response to increasing doses of methacholine vs. PBS; HDM/HDM (dark orange), HDM/HDM VacA (green) and HDM/PBS mice (light orange). Asterisks indicate difference between HDM/HDM and HDM/HDM VacA. **(C)** Cellular composition of bronchoalveolar lavage (BAL): total cell count (Tcc), macrophages (Mac), lymphocytes (Lymph), neutrophils (Neutros) and eosinophils (Eos); HDM/HDM (black), HDM/HDM VacA (dark gray) and HDM/PBS mice (white). **(D)** Inflammation in lung tissue: pictures show representative sections of each indicated group (x100). Scatter plot: inflammation score in HDM/PBS, HDM/HDM and HDM/HDM VacA animals. **(E)** Mucus-producing cells: pictures show representative sections from each group (x200). Scatter plot: number of mucus-producing cells/mm basement membrane in HDM/PBS, HDM/HDM and HDM/HDM VacA animals. **(F)** VacA treatment affects immunoglobulin subtypes. Graphs: house dust mite (HDM)-specific immunoglobulin (Ig)E, -IgG1, -IgG2b titers and ratio of HDM IgG1 to IgG2b in HDM/phosphate-buffered saline (PBS), HDM/HDM and HDM/HDM VacA animals. **(B, C)** results from five independent experiments, n=15–20 per group. **(D–F)** each symbol represents one animal. **(D, E)** results from four independent experiments, n=12–16 per group. **(F)** Results from five independent experiments, n=16–21 per group. Analysis of variance: *p<0.05, **p<0.01, ***p<0.001; ns, not significant.

Induction of house dust mite (HDM)-dependent allergic airway disease was performed using a previously described model (15–17). Briefly, on day 0, isoflurane-anesthetized mice were sensitized using intranasal application of 1 µg HDM protein [Greer Laboratories, USA] dissolved in 50 µL phosphate-buffered saline (PBS). Animals were then challenged with 10 µg HDM protein in 50 µL PBS given intranasally to isoflurane-anesthetized mice from day 7–11. Based on experience from previous studies, the following VacA treatment regimen was implemented: animals received 20 µg of VacA dissolved in 100 µL PBS *via* intraperitoneal (i.p.) injection on days 6, 7, 9 and 11 (**Figure 1A**). In these experiments, either an active strep-tagged oligomeric s1m1 type VacA (VacA) or an inactive strep-tagged VacA mutant protein (VacAΔ6-27; mutVacA) purified from modified forms of *H. pylori* strain 60190, or a C-terminal 6xHistidine-tagged s1m1 recombinant VacA protein derived from the same *H. pylori* strain (rVacA; provided by GBC-HpVac [Geneva, Switzerland]) was used. VacA and mutVacA were purified as described previously (18–20), and rVacA was purified using similar standard purification steps as follows: the rVacA was captured in a NiNTA affinity chromatography step, followed by size-exclusion chromatography using a S-200 resin as a polishing step. The resulting protein was >98% pure, as judged by analytical gel filtration and SDS-PAGE (not shown).

**Figure 2:**
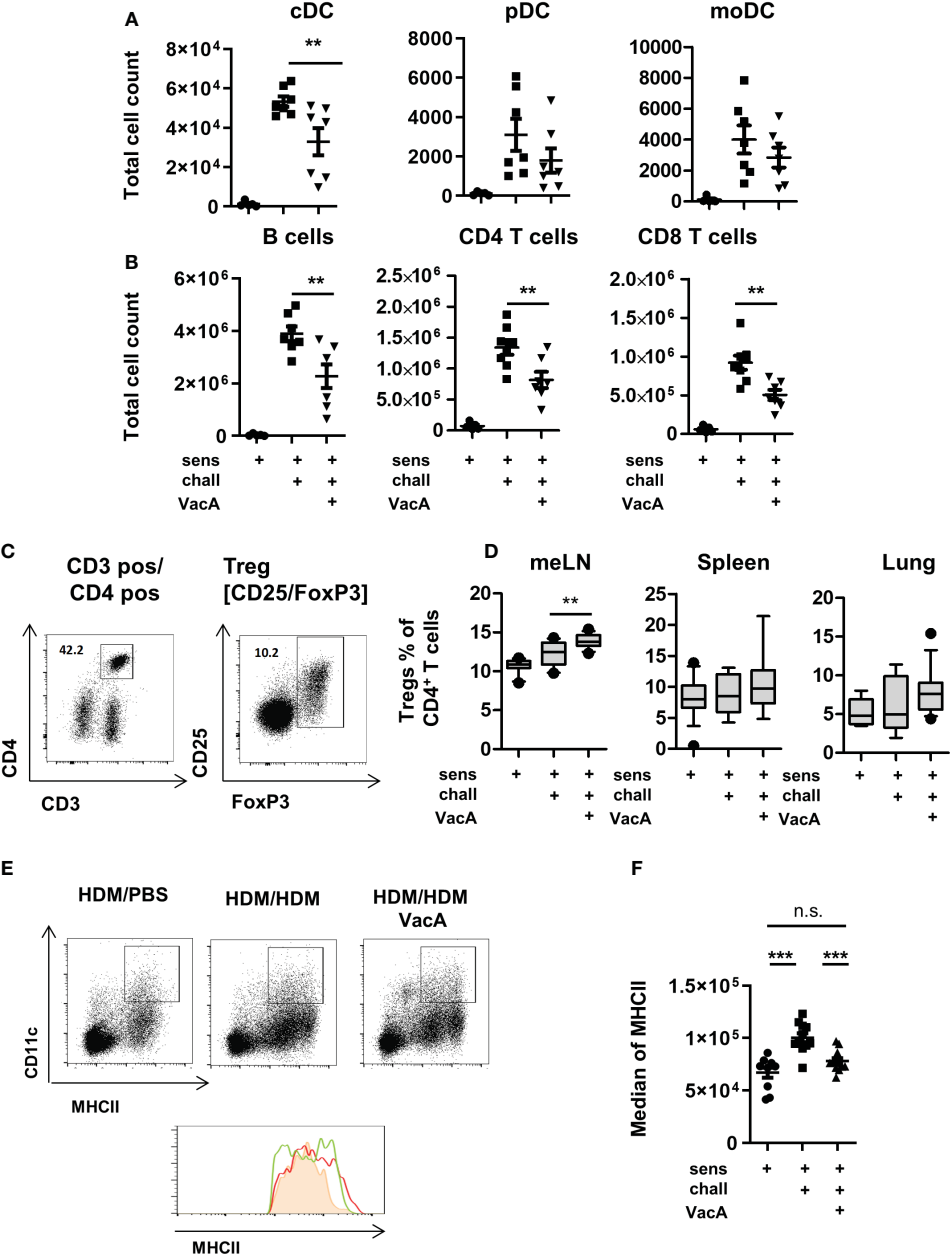
VacA treatment affects immune phenotype. Total cell counts of **(A)** conventional dendritic cells (cDC), plasmacytoid DC (pDC) or monocyte-derived inflammatory DC (moDC) and **(B)** B cells. CD4+ and CD8+ T cells in lung draining lymph nodes (tLN) of house dust mite (HDM)/phosphate-buffered saline (PBS), HDM/HDM and HDM/HDM VacA animals. (C, D) VacA treatment increases proportion of Tregs. **(C)** Tregs were characterized by the expression of FoxP3 within the population of CD3+/CD4+ T helper cells. **(D)** Boxplots (Whiskers 10-90 percentile) show percentage of Tregs within the population of CD4+ T cells in mesenteric lymph nodes (meLN), Spleen and tLN of HDM/PBS, HDM/HDM and HDM/HDM VacA animals. **(E, F)** DC in VacA-treated animals showed reduced major histocompatibility complex II (MHCII) expression in draining lymph nodes. **(E)** Representative dot plots show MHCII/CD11c expressing DC in draining lymph nodes of HDM/PBS, HDM/HDM and VacA-treated HDM/HDM mice. Histogram shows MHCII expression in HDM/PBS (shaded orange), HDM/HDM (red line) and HDM/HDM VacA animals (green line). **(F)** Graph shows median MHCII expression on DC in the draining lymph nodes of HDM/PBS, HDM/HDM and HDM/HDM VacA-treated mice. Each point represents one animal. **(A, B)** results from two independent experiments, n=5–7 per group. **(C, D)** results from 3–5 independent experiments, n=10–21 per group. **(E, F)** results from three independent experiments, n=9–12 per group. Analysis of variance: *p<0.05, **p<0.01, ***p<0.001.

**Figure 3:**
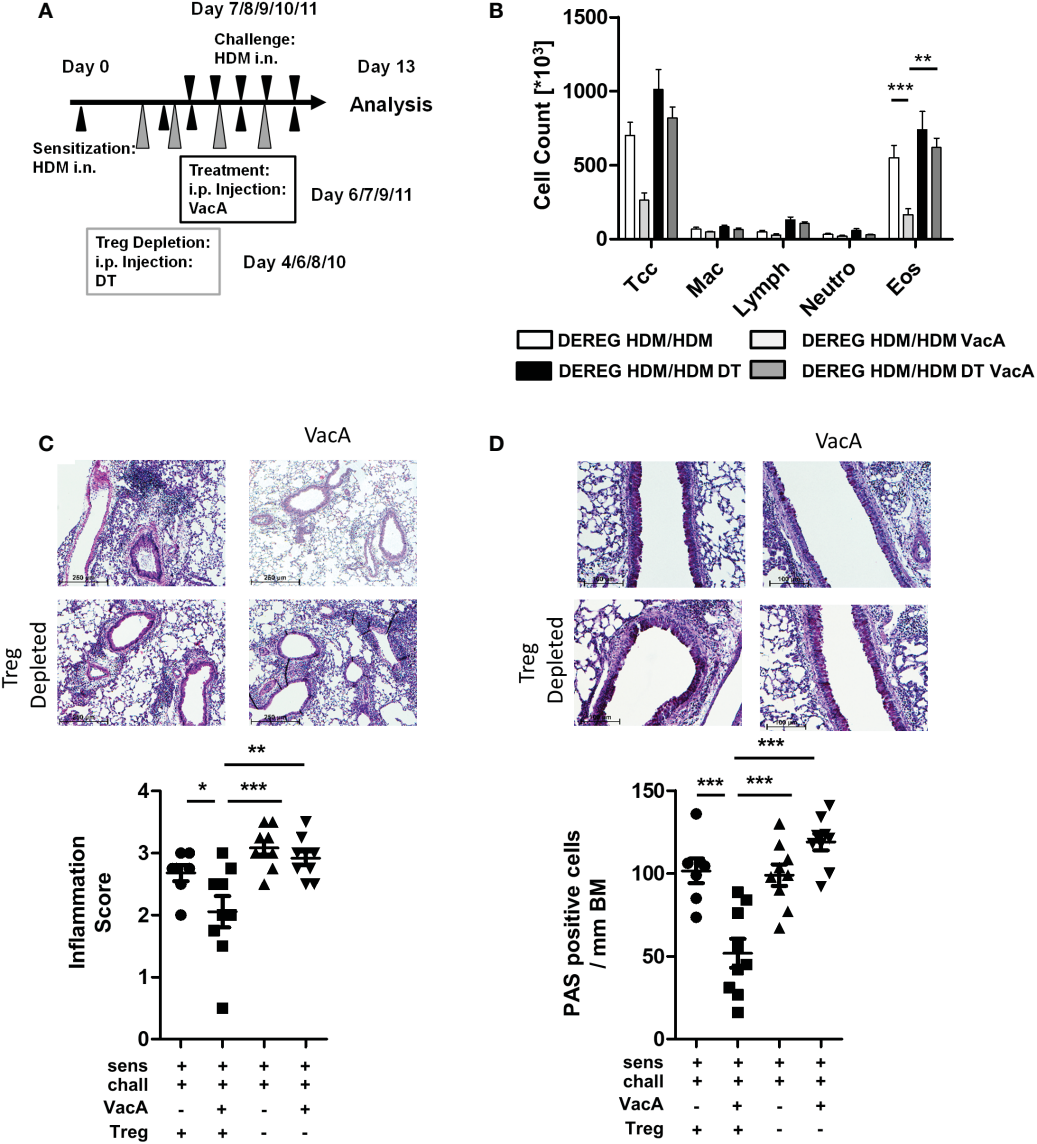
Depletion of Tregs in VacA-treated DEREG mice abolished therapeutic effects in the house dust mite (HDM)-specific allergic lung disease model. **(A)** Tregs were depleted by repeated intraperitoneal administrations of diphtheria toxin (DT) on days 4, 6, 8 and 10. **(B)** Cellular composition of bronchoalveolar lavage (BAL): total cell count (Tcc), macrophages (Mac), lymphocytes (Lymph), neutrophils (Neutro) and eosinophils (Eos); DEREG HDM/ HDM (white), DEREG HDM/HDM VacA (light gray), DEREG HDM/HDM DT (black) and DEREG HDM/HDM DT VacA (dark gray) mice. **(C)** Inflammation in lung tissue: pictures show representative sections from each indicated group (x100). Scatter plot: inflammation score in DEREG HDM/HDM, DEREG HDM/HDM VacA and DEREG HDM/HDM DT and DEREG HDM/HDM DT VacA mice. **(D)** Mucus-producing cells: pictures show representative sections from each group (x200). Scatter plot: number of mucus-producing cells/mm basement membrane in DEREG HDM/HDM, DEREG HDM/HDM VacA and DEREG HDM/HDM DT and DEREG HDM/HDM DT VacA mice. Results from three independent experiments, n=5-9; Analysis of variance: *p<0.05, **p<0.01, ***p<0.001

Asthma phenotype and immunological readouts were assessed on day 13 (**Figures 1**, **2**). To determine the functional role of Tregs in VacA-mediated immune suppression, DEREG mice and a modified version of the previously described allergic airway disease/VacA treatment model were used. In addition to the HDM sensitization/challenge and treatment with VacA, wild type (WT) and DEREG mice received 30 ng/g DT dissolved in 100 µL PBS i.p. on days 4, 6, 8 and 10 (**Figure 3A**) to deplete Tregs and control side effects.

#### 2.3.2 Therapeutic HDM-specific murine allergic airway disease model

**Figure 4:**
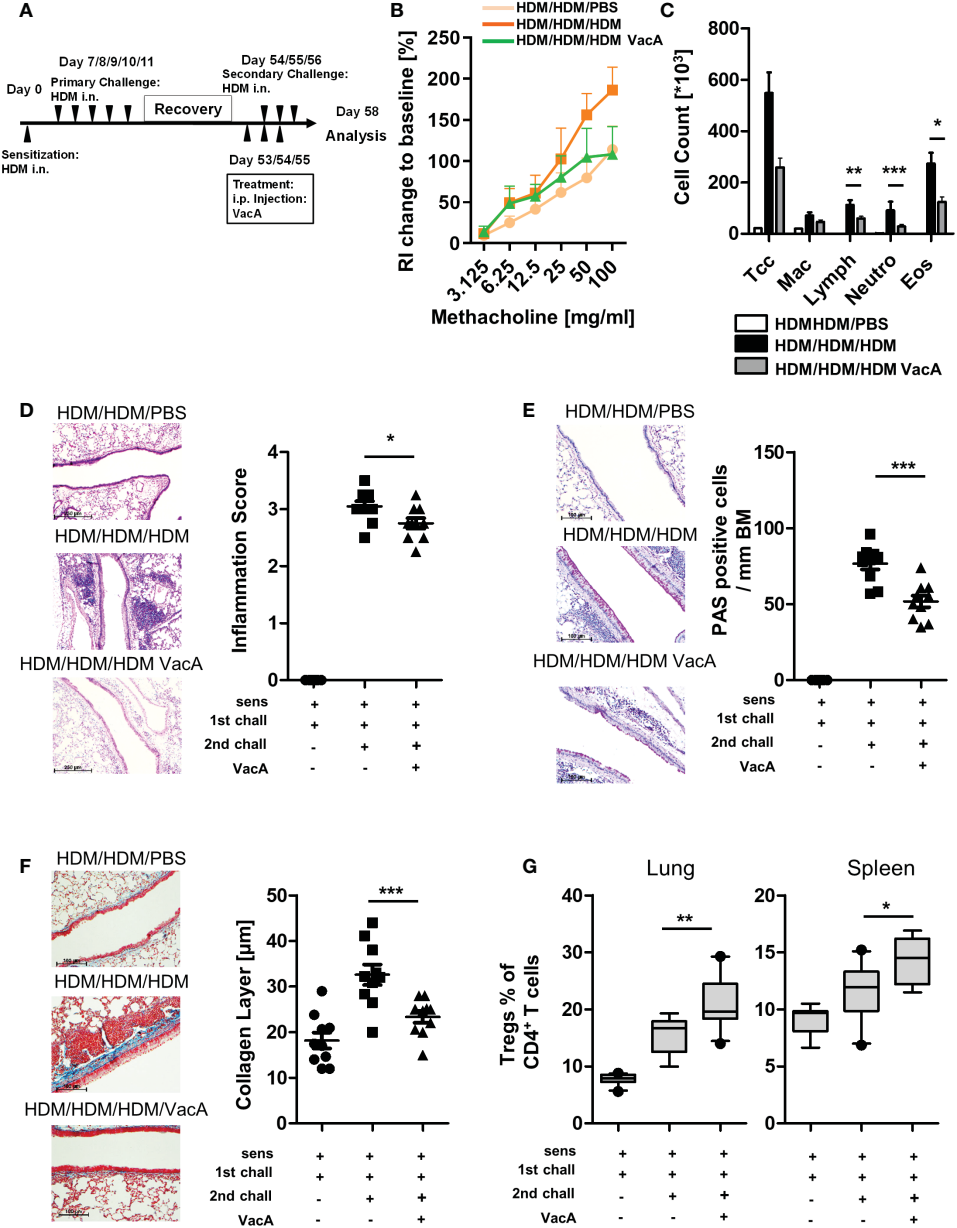
VacA attenuates asthmatic reactions in mice with already established lung disease. **(A)** secondary allergen challenge was performed after house dust mite(HDM) sensitization and challenge, and 6 weeks’ rest. VacA treatment was given during the secondary challenge. **(B)** Change of airway resistance (RI):percentage change in airway resistance in response to increasing doses of methacholine vs. PBS; HDM/HDM/HDM (dark orange), HDM/HDM/HDM VacA(green) and HDM/HDM/PBS mice (light orange) **(C)** Cellular composition of BAL: number of total cells (Tcc), macrophages (Mac), lymphocytes (Lymph),neutrophils (Neutro) and eosinophils (Eos); negative control (HDM/HDM/PBS [white]), positive control (HDM/HDM/HDM [black]) and HDM/HDM/HDMVacA (dark gray). **(D)** Inflammation in lung tissue: pictures show representative sections from each indicated group (x100). Scatter plot: inflammationscore in HDM/HDM/PBS, HDM/HDM/HDM and HDM/HDM/HDM VacA mice. **(E)** Mucus-producing cells in lung airways: pictures show representativesections from each group (x200). Scatter plot: number of mucus-producing cells/mm basal membrane of HDM/HDM/PBS, HDM/HDM/HDM and HDM/HDM/HDM VacA mice. **(F)** Subepithelial collagen deposition: pictures show representative sections from each group (x200). Scatter plot: averagedsubepithelial collagen layer thickness in μM in HDM/PBS, HDM/HDM and HDM/HDM VacA animals. **(G)** VacA treatment increases Treg frequencies.Boxplots (Whiskers 10-90 percentile) show proportion of Tregs within the population of CD4-positive T cells in the lung and spleen of HDM/HDM/PBS,HDM/HDM/HDM and HDM/HDM/HDM VacA animals. Each symbol represents one animal. Data are results from two independent experiments, n=10 per group. Analysis of variance: *p<0.05, **p<0.01, ***p<0.001.

The therapeutic effectiveness of VacA was determined in a modified model of allergic airway disease. To induce allergic airway disease, animals were sensitized and challenged with HDM as already described. After a resting phase of 6 weeks, animals received a secondary HDM challenge on days 54, 55 and 56 (10 µg HDM protein/50 µL PBS). Therapeutic VacA treatment (20 µg/100 µL PBS i.p.) was administered on days 53, 54 and 56. Asthma phenotype and immunological readouts were assessed on day 57 (**Figure 4A**). Three different experimental groups were compared: 1. negative control (sensitized and 1^st^ challenged to HDM, 2^nd^ challenge with PBS – HDM/HDM/PBS), 2. positive control (sensitized and 1^st^ challenged and 2^nd^ challenge with HDM – HDM/HDM/HDM) 3. VacA treatment group (sensitized and 1^st^ challenged and 2^nd^ challenge with HDM + treatment with VacA – HDM/HDM/HDM VacA) (Details in **Figure 4A**).

Each of these experiments was performed at least twice (actual animal numbers are indicated in the figure legends).

### 2.4 Assessment of asthma hallmarks

Lung function, differential bronchoalveolar lavage (BAL) cell counts, and histological analysis of lung slides were performed to analyze the asthma phenotype.

#### 2.4.1 Analysis of lung function

Invasive measurement of lung function in response to increasing doses of methacholine (MCh, Sigma-Aldrich; mg/mL PBS) was performed on anesthetized, intubated mechanically ventilated mice using the Fine Point system (FinePointe RC units; Data Sciences International, New Brighton, MN). The percentage change in maximum resistance after each dose of MCh compared with PBS nebulization was calculated.

#### 2.4.2 Analysis of BAL

Lungs were lavaged with 1 mL ice-cold PBS *via* an intratracheal tube. BAL cell counts were determined. Differential cell counts of at least 200 counted cells for macrophages, lymphocytes, neutrophils, and eosinophils were performed on cytocentrifuged preparations stained with Hemacolor-Set (Merck). From these, percentages and absolute cell counts were calculated.

#### 2.4.3 Analysis of lung histology

After preparation of the left lung lobe for flow cytometric analysis, right lobes were fixed by inflation and immersion in Histofix (Roth) and embedded in paraffin. Tissue sections were prepared and stained with hematoxylin/eosin (HE) to analyze tissue inflammation, or with combined Periodic Acid Schiff (PAS)/HE staining to identify mucus-producing goblet cells. Analysis was performed as described previously (21).

Airway inflammation was scored semi-quantitatively on HE slides. For this, five randomly selected areas were scored by two experienced observers blinded to experimental groups. Inflammation was scored on a scale from 0 to 4 (detailed description in [21)]. The score is based on the following inflammatory conditions: 0, all airways and vessels are free of inflammatory infiltrates; 1, some infiltrates are detectable around airways and vessels, and are up to two layers in thickness; 2, the majority of airways and vessels show infiltrates with thickness of up to two layers, with more layers found only occasionally; 3, the majority of airways and vessels show inflammatory infiltrates, and many layers are thicker than two layers, ranging from 3–8 layers; 4, all airways and vessels are surrounded by thick layers of inflammatory cells.

PAS-positive goblet cells were quantified per millimeter of basal membrane on at least three different representative airways on PAS-stained slides. To analyze subepithelial collagen deposition, Masson Trichrome staining was performed on lung slides from the secondary challenge model. At least three different airways were randomly selected, and thickness of the collagen layer was measured at five different areas of each airway. Mean collagen layer thickness of each airway was calculated and then averaged between all airways.

### 2.5 Preparation of single cell suspensions from different tissues

#### 2.5.1 Lungs

After perfusion was performed, *via* the right ventricle, ligated left lung lobes were eviscerated, minced and transferred into 50-mL tubes. Collagenase type I (0.5 mg/mL; catalog no. C9891, Sigma) was added and after incubation in a shaking water bath for 45 minutes at 37°C, cells were passed through a cannula (20G 0.9 mm x 40 mm) in a 10-mL syringe and then through a 70-μm cell strainer at least three times to obtain a single cell suspension. Erythrocytes were lysed, and cell counts were determined.

#### 2.5.2 Lymph nodes (mesenteric lymph node; tracheal lymph node)

Lymph nodes were carefully grinded between the slides, cells were washed into Hanks’ balanced salt solution (HBSS) and transferred through a 70 µm cell strainer into a tube. Cell counts were determined.

#### 2.5.3 Spleen

Organs were minced and transferred through a 70 µm cell strainer into a 50 mL tube. Red blood cell (RBC) lysis was performed as already described, cells were washed, and cell counts determined.

All single cell preparations were adjusted to 1x10^7^ cells/mL IMDM containing 10% fetal calf serum (FCS; PAA Laboratories), 1% Pen/Strep (Gibco; 10,000 U/mL penicillin; 10,000 µg/mL streptomycin) and stored on ice until further processing.

### 2.6 Flow cytometric analysis

Prepared single cell suspensions (5x10^5^ cells) were plated in 96 well plates (TC Plate 96 Well Suspension, R [Sarsted]). Unspecific antibody binding was blocked with 0.5 μL of Fc receptor–blocking antibodies [TruStain FcX™ (anti mouse CD16/32) (Isotyp: Rat IgG2a, λ clone: 93 [BioLegend])]. Antibody panels defined in **Supplementary Table 1** were used to analyze DC and B cells (murine DC/B cell analysis) and T cells (murine Treg analysis panel). To analyze Tregs, intracellular staining against the transcription factor FoxP3 was performed on surface-stained T cells using the FoxP3/Transcription Factor Staining Buffer Set [eBioscience]. All cells were finally fixed with Fixation Buffer [PBS + 2% paraformaldehyde [Sigma-Aldrich] and analyzed.

To determine T cell populations following analysis strategy was used. First, debris and doublets were excluded based on size and shifting properties seen by analysis of FSC-Height vs. FSC-Area. autofluorescent cells, positive cells in an empty channel were excluded. Based on the expression of CD8/CD3 cytotoxic T cells were differentiated from CD3/CD4 positive T Helper cells. Tregs were then defined as FoxP3 positive cells within the population of CD3/CD4 positive cells (22). Treg subpopulations were further subdivided in CD25^+^ cell and CD25^-^ cells (**Supplementary Figure 1B**) (23).

To determine DC subpopulations and B cells, first debris and doublets were excluded, and then B cells were identified as CD19-positive cells. Within the remaining cells, DC were characterized as CD11c/MHCII-positive cells. Subpopulations like pDC (CD11c^+^/MHCII^+^/B220^positive^), cDC (CD11c^+^/MHCII^+^/Ly6c^negativ^B220^negative^), inflammatory DC (CD11c^+^/MHCII^+^/Ly6c^positive^B220^negative^), were identified using the indicated marker combination (**Supplementary Figure 1A**).

The absolute cell count of the individual cell populations was calculated using the relative proportions of the individual cell populations and the absolute cell counts of the organ determined during the experiments.

Measurements were performed on a CYTOFLEX flow cytometer (Beckman Coulter), FCS files were analyzed and graphics were generated using FlowJo Software (version 10.6.1).

### 2.7 HDM-specific ELISA

To analyze HDM-specific immunoglobulins (Ig - IgG1; IgG2b and IgE) serum was collected at day 13 in the HDM-specific murine allergic airway disease model. Enzyme-linked immunosorbent assay (ELISA) plates (Microplate, 96 Well, PS, F-Bottom, clear, Microlon^®^, High Binding [Greiner bio-one]) were coated with HDM coating solution (3,125 µg/mL HDM in 50 µL coating buffer (100 mM carbonate-bicarbonate, pH 9.5) for 24 hours. Plates were blocked with 300 µL PBS containing 2% bovine serum albumin (BSA; SIGMA) and incubated for 1 hour. After three washing steps, 50 µL of adequately diluted serum duplicates were applied to the plate and serial diluted over four steps and incubated for 2 hours (IgE dilutions 1:10/20/40/80, IgG1 dilutions 1:200/400/800/1600, IgG2b dilutions 1:100/200/400/800). After removal of the serum and three washing steps, biotin conjugated primary antibodies for IgG1 (Biotin Rat Anti-Mouse IgG1, clone: A35-1, BD Pharmingen™), IgG2b (Biotin Rat Anti-Mouse IgG2b, clone: R12-3, BD Pharmingen™) or IgE (Biotin Rat Anti-Mouse IgE, clone: R35-118, BD Pharmingen™) were applied in 1% BSA in PBS and incubated for 1 hour. After three washing steps, streptavidin conjugated horseradish peroxidase (BD Pharmingen™) was applied and incubated for 1 hour and removed with three washing steps. For the color detection reaction, the tetramethylbenzidine (TMB) Substrate Reagent Set from BD OptEIA™ was added; colorimetric reaction was stopped by addition of 1M sulfuric acid. Optical density (OD) was measured with plate reader Microplate Reader (BIO-RAD iMark™). The antibody titer was defined as the reciprocal serum dilution yielding an absorbance reading of OD=0.2 after linear regression analysis.

### 2.8 Analysis of impact of VacA on murine bone marrow-derived DCs

DCs were generated from bone marrow of C57BL/6 mice as described previously by Beckert et al. (24).

**Figure 5:**
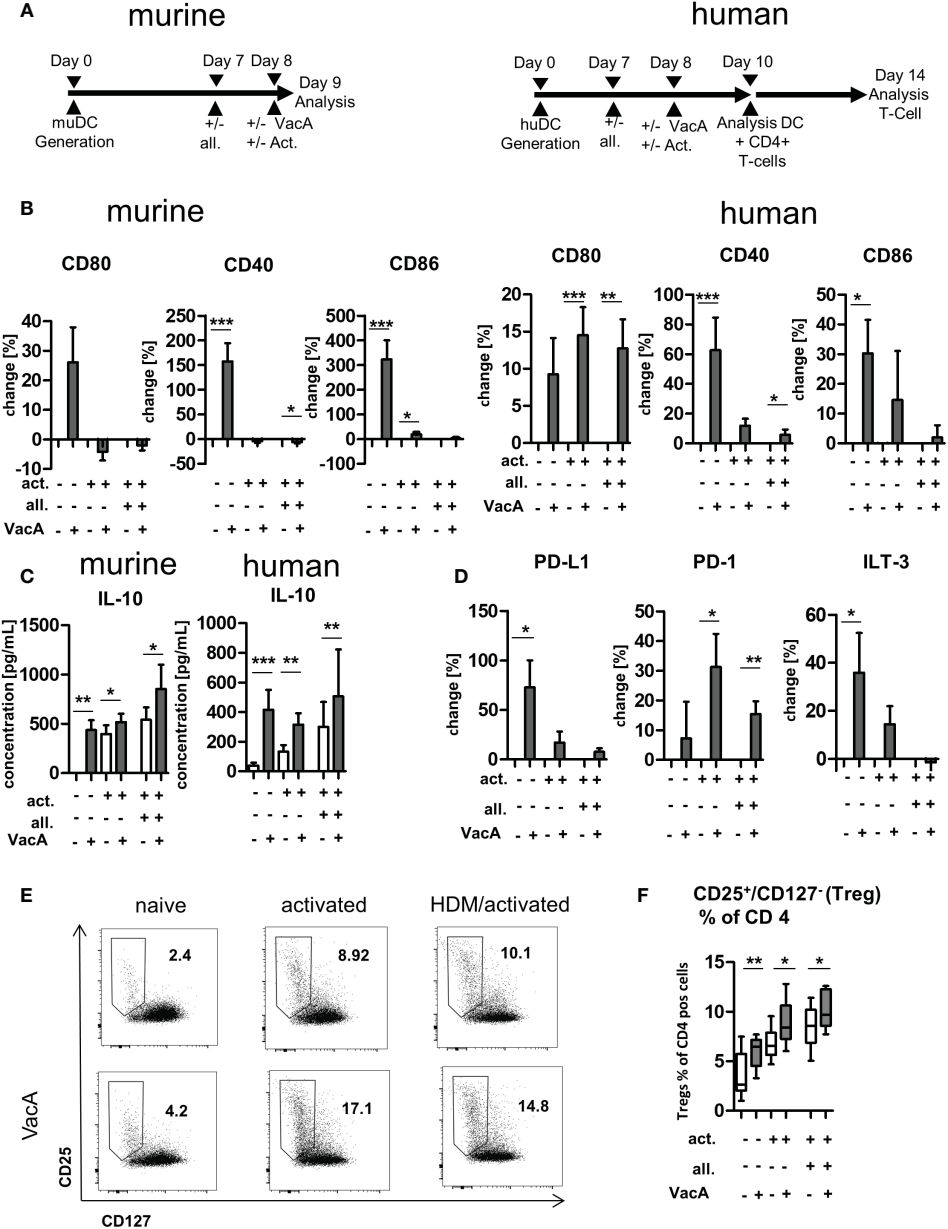
VacA modulates expression of stimulatory and inhibitory co-receptors and cytokines in murine and human dendritic cells (DC) and induces humanregulatory T cells (Tregs). **(A)** Treatment scheme: murine bone marrow DC (BMDC)/human DC: after 7 days of differentiation DC were treated +/-allergen (all), then DC activation (act.) was performed and treatment with VacA was given. Cultivation of human DC with autologous T cells wasperformed from day 10–14. **(B)** Change in expression of the indicated surface marker after treatment with VacA versus naïve, activated, or activated andallergen-supplemented murine and human DC. **(C)** Supernatant concentrations of interleukin (IL)-10 in naïve, activated, and activated and allergensupplementedmurine and human DC cultures with or without VacA. **(D)** Change in expression of the indicated surface marker with inhibitory capacityversus VacA treatment on the surface of naïve, activated, or activated and allergen-supplemented human DC (murine, n=14; human, n=10). AutologousT cells were cultured with the differentially activated and VacA-treated DC and the proportion of regulatory T cells was then examined. **(E)** Dot plots:regulatory T cells were identified by gating CD25+/CD127- cells within the population of CD4+/CD3+ T helper cells. Dot plots show representativeexamples for CD25/CD127 staining of naïve, activated, and activated and allergen-supplemented human DC/T cell cultures with or without VacAtreatment. Gate and proportion of Tregs is highlighted. **(F)** Percentage of Tregs within the CD4+ T cell population. VacA treatment significantly increasedTregs in all analyzed culture conditions (n=10). Wilcoxon signed rank test: *p<0.05, **p<0.01, ***p<0.001.

On day 8, 1x10^5^ cells were seeded into a 96-well round bottom plates and three different conditions were performed: a) naïve (cultured without any further stimulus); b) activated, cultured in presence of lipopolysaccharide (LPS [1 µg/mL; Sigma, *E.coli* O111:B4]); c) activated and allergen-treated, cultured following a standardized protocol in presence of the allergen ovalbumin (OVA; [EndoGrade^®^ (purity >98%, endotoxin c: <1 EU/mg)]) loading (5 µg/mL) on day 7 and activated with LPS (1 µg/mL added at day 8). In each condition, VacA was applied without any further modifications at a concentration of 10 µg/mL, one hour before activation was performed (**Figure 5A**).

The activation state of naïve, activated and activated cells additionally treated with allergen was analyzed by flow cytometry 24 hours later. Using the antibodies listed in **Supplementary Table 2** (murine DC activation panel), cells were stained as already described and the following strategy was used to characterize DC. Following exclusion of doublets and dead cells, DCs were identified by the expression of CD11c and MHCII. Expression of the activation markers CD80, CD86, CD40 was analyzed. Mean expression intensity was determined using FlowJo. To better compare different experiments with each other, the percentage change in expression between VacA treatment and the corresponding comparison group was calculated. Supernatants of DCs were stored at –20°C.

### 2.9 Analysis of impact of VacA on PBMC-derived human DC and their interaction with autologous T cells

Human DCs were differentiated from monocytes isolated from PBMC using a modified version of a previously published protocol (25). In brief, after isolation of PBMC using BioColl^®^ (Bio&Sell), monocytes were isolated from PBMC with a magnetic bead sorting system from Miltenyi (Pan Monocyte Isolation Kit, human), as recommended by the manufacturer. In a Nunc EasyFlask 75 cm^2^ Nunclon™ Delta Surface cell flask, 10^7^ cells/10mL were seeded and incubated (5% CO_2_ at 37°C) for seven days in DC medium (X-Vivo-15 medium [Lonza]) supplemented with granulocyte-macrophage colony-stimulating factor (800 U/mL; MiltenyiBiotech), interleukin (IL)-4 (1000 U/mL; PeproTech), 2% autologous serum, and 1% penicillin streptomycin (Gibco). After 4 days, the same amount of medium was added to the cells. After 7 days, cells were collected with PBS/EDTA buffer (PBS, 2 mM EDTA pH 7.4–7.9) and 10^6^ cells/mL were plated into 96-well round bottom plates in DC medium. The impact of VacA was analyzed in three different conditions: a) naïve cells, cultured without any further stimulus; b) activated cells, cultured in presence of an activation cocktail containing 30 ng/mL IL-1β (Miltenyi), 30 ng/mL tumor necrosis factor (TNF)-α (Miltenyi Biotech) and 3 µg/mL prostaglandin-D2 (Cayman Chemical) for 48 hours from day 8–10; and c) allergen-treated/-activated cells, incubated with HDM (6.5 µg/mL Greer) at day 7 and treated with the activation cocktail from day 8–10. In all groups, VacA (10 µg/mL) was added at day 8 without any further modifications (**Figure 5A**).

Impact on activation state was analyzed *via* flow cytometry at day 10 using antibodies listed in **Supplementary Table 2** (human DC activation panel) and the following gating strategy. Staining of dead cells, blocking of FcγR (Human TruStain FcX™; BioLegend) and staining with specific antibodies was performed as described above. Following exclusion of doublets and dead cells, moDC were identified by expression of HLA-DR and CD11c. Expression of surface molecules with immune-activatory (ITAMs) and inhibitory (ITIMs) properties were analyzed. Comparable to the mouse model, mean expression of the ITAMs CD80, CD40 and CD86 were measured. Additionally, surface expression of the ITIMs programmed death ligand (PD-L) 1, programmed death (PD)-1 and ILT3 was measured (**Supplementary Figure 1B**). To better compare different experiments with each other, the percentage change in expression of the MFI of the corresponding molecule was compared between VacA treatment and the corresponding comparison group. moDC supernatants were stored at –20°C.

To analyze the impact of VacA on Treg modulation, differentially treated moDC were cultivated with PBMC-derived autologous T cells. PBMC were isolated using BioColl^®^. CD4-positive T cells were isolated as per the manufacturer’s instructions (CD4+ T Cell Isolation Kit, human, Miltenyi). T cells were cultured with moDCs (5:1 ratio) for 4 days in Xvivo supplemented with 1% PBS and 2% autologous medium (**Figure 5A**). To identify Tregs, doublets and dead cells were excluded, CD4^+^ T cells were chosen, and Tregs were characterized as CD127^-^/CD25^+^ cells as described previously (26, 27). For detailed antibody information, see **Supplementary Table 2** (human Treg analysis panel). The ratio of Tregs analyzed using FlowJo and expression were compared between all treatment groups.

All experiments with human samples were performed in accordance with the ethics vote 18-8069-BO approved by the medical faculty of the University Duisburg-Essen, Germany.

### 2.10 Cytokine detection in DC supernatants

To detect production of IL-10 and other cytokines, the LEGENDplex™ HU Th Cytokine Panel (12-plex) or the LEGENDplex™ MU Th Cytokine Panel (12-plex) assays were performed with supernatants from human moDC or murine *in vitro* DC assays, respectively. Assays were performed and cytokine concentrations were calculated according to the manufacturer’s instructions.

### 2.11 Statistical analysis

Values for all measurements are expressed as mean ± standard error of the mean (SEM). Analysis of variance (ANOVA) was used to determine all between-group differences. To compare differences between two groups, an Anderson–Darling test was first performed to control gaussian approximation, and then an appropriate test (unpaired Student t-test or Mann–Whitney U test) was used for analyses. The Wilcoxon signed ranked test was used for comparisons in *in vitro* experiments. The p-value cutoff for statistical significance was set at 0.05.

## 3 Results

### 3.1 Treatment with *H. pylori*-derived VacA ameliorates allergic airway disease

HDM sensitization and challenge (**Figure 1A**) resulted in development of airway hyperreactivity, airway inflammation and goblet cell metaplasia, all of which were significantly attenuated by treatment with VacA (**Figures 1B–E**). No suppression of allergic airway disease was detectable when animals were treated with a mutant VacA protein (VacA Δ6-27) (**Supplementary Figure 2**).

Treatment of animals with rVacA also suppressed airway inflammation and goblet cell metaplasia (**Supplementary Figure 3**), supporting the therapeutic effect of VacA in the allergic airway disease model.

In sensitized and challenged animals there were no differences between VacA-treated and placebo-treated animals with respect to changes in bodyweight over time, signs of peritoneal inflammation and animal distress scores (data not shown).

### 3.2 Treatment with VacA modulates the composition of allergen-specific immunoglobulin subtypes

Sensitization and challenge with allergen increased production of allergen-specific IgE and IgG1 compared to mice treated with PBS during the challenge phase (**Figure 1F**). Treatment with VacA did not affect serum IgE, and was associated with a small, statistically insignificant decrease in IgG1 levels compared with sensitized and challenged animals. However, animals treated with VacA showed an increase in serum levels of allergen-specific IgG2b, resulting in a significant reduction in the IgG1/IgG2b ratio compared with the positive control.

### 3.3 Treatment with VacA modulates the immune phenotype

Animals treated with VacA showed significantly reduced numbers of conventional (c)DCs, B cells, and CD4- and CD8-positive T cells in the lung draining lymph node (**Figures 2A, B**). Compared with cDCs, there were no significant detectable differences in the number of plasmacytoid and inflammatory monocyte-derived DCs between animals in the positive control group and those in the VacA-treated group (**Figure 2A**).

Treatment with VacA slightly but significantly increased the frequencies of CD4^+^/FoxP3^+^ Tregs in mesenteric lymph nodes (**Figures 2C, D**). In spleen, lung and lung draining lymph node was no statistically significant between untreated and VacA treated animals detectable. (**Figure 2D**).

To evaluate if VacA affects Treg subset, CD25 positive and negative FoxP3 positive Tregs were distinguished. None of the analyzed organs demonstrated a significant difference in the composition of these two subpopulations between VacA treated and untreated groups (**Supplementary Figure 4**).

Animals treated with rVacA showed comparable induction of Tregs in mesenteric lymph nodes (meLNs) and demonstrated a significantly increase of Tregs in the spleen as well (**Supplementary Figure 3D**). In comparison to untreated sensitized and challenged animals demonstrated DCs in the lung draining lymph nodes of animals treated with VacA a slightly reduced expression of MHCII molecules on their surface (**Figures 2E, F** and **Supplementary Figure 1C**).

### 3.4 Tregs play a pivotal role during VacA-mediated immune suppression

To assess the role of Tregs in mediating the protective effects of VacA on allergen-induced airway disease, the same treatments as shown in **Figure 1** were performed in DEREG mice (13), which allows the depletion of Tregs by injecting DT (**Figure 3A**).

Similar to the previous experiments, sensitized and challenged DEREG mice developed eosinophilia in BAL (**Figure 3B**), inflammatory infiltrates in lung tissue (**Figure 3C**), and goblet cell metaplasia (**Figure 3D**). Treatment with VacA reduced BAL eosinophils and improved lung histology. Depletion of Tregs *via* administration of DT in sensitized and challenged DEREG mice did not affect the inflammatory phenotype. However, depletion of Tregs in VacA-treated mice abolished VacA-mediated immune suppressive effects, and animals developed eosinophilia in BAL, lung inflammation and goblet cell metaplasia that was comparable to the untreated positive control (**Figures 3B–D**).

DEREG animals receiving DT showed a lower proportion of GFP-positive Tregs in meLN, spleen and tLN (**Supplementary Figure 5**). There was no difference in the number of inflammatory cells in sensitized and challenged WT animals, and sensitized and challenged WT animals treated with DT (data not shown).

### 3.5 Effectiveness of VacA in a therapeutic model

To further analyze the effectiveness of VacA as a therapeutic molecule, a secondary challenge model was utilized. In this setting, treatment of VacA was applied to animals with already developed allergic airway disease during a secondary, repeated exposure to the allergen (**Figure 4A**). In this model, treatment with VacA ameliorated the lung function decline (**Figure 4B**) and attenuated the inflammatory phenotype of allergic airway disease, including reducing the numbers of lymphocytes, neutrophils and eosinophils in BAL (**Figure 4C**), and attenuating both inflammation (**Figure 4D**) and goblet cell metaplasia in the lung (**Figure 4E**). Induction of subepithelial collagen deposition, a clear sign of airway remodeling, was significantly reduced in mice treated with VacA (**Figure 4F**). Furthermore, there was a significant increase of Tregs in the spleen and lung after treatment with VacA (**Figure 4G**).

### 3.6 VacA exposure drives tolerogenic reprogramming of murine and human DC

To characterize the influence of VacA on DC and T cells in more detail and to draw first conclusions about its effectiveness in humans, murine and human cell culture experiments were carried out. Murine and human DCs were generated from bone marrow and PBMCs, respectively, and the effects of VacA were analyzed alone and in combination with allergen exposure (**Figure 5A**). Application of VacA to naïve murine and human DC resulted in upregulation of the co-stimulatory molecules CD40, CD80 and CD86 (**Figure 5B**). Adding VacA to activated cells or activated cells treated with allergen did not further increase the expression of these markers (**Figure 5B**). The application of VacA strongly induced the production of the immune suppressing cytokine IL-10 in both murine and human DCs. Increased concentrations of IL-10 after VacA exposure were detectable in all analyzed conditions (**Figure 5C**). Naïve human DCs treated with VacA showed increased surface expression of PD-L1 and ILT3; these markers only tended to be increased in VacA-treated activated and allergen treated activated cells. However, these cells showed increased expression of PD-1 after VacA treatment compared with the respective control (**Figure 5D**).

Exposure of human T cells to autologous DCs treated with VacA resulted in the induction of CD127^-^/CD25^+^ CD4^+^-positive T cells in all investigated conditions (**Figures 5E, F**). In addition, flow cytometric analyses of dead cells did not reveal any VacA-mediated cytotoxic effects on the *in vitro* cultures (**Supplementary Figure  6**).

## 4 Discussion

In the present study, we report that the systemic therapeutic administration of purified VacA (either the form secreted by *H. pylori* or a recombinant version) reduced allergic airway disease, as shown by decreased airway inflammation, tissue inflammation, PAS-positive cells and airway reactivity. Unlike previous studies that only showed a prophylactic efficacy of VacA after long-term treatment started shortly after birth, the current data demonstrate the potential therapeutic efficacy of this approach in asthma for the first time (9, 19). In both acute and therapeutic HDM-specific models of allergic airway disease, treatment of adult mice during the primary and secondary allergen challenge phase was associated with a reduction of the asthma phenotype. Beneficial effects on lung function, the inflammatory reaction in the lungs and BAL, and on restructuring processes in the lungs could be observed. The results of this study also suggest that VacA can modulate the function of both murine and human DCs in a similar manner. Furthermore, the VacA-mediated induction of Tregs in human *in vitro* cultures supports observations from the murine *in vivo* experiments and could thus represent a first indication of the effectiveness of VacA in humans.

The current results are consistent with previous studies investigating the activity of crude *H. pylori*-derived bacterial lysates containing all kinds of bacterial compounds in allergic airway disease (28, 29). Interestingly, treatment with pure VacA appeared to be more effective than the *H. pylori* lysate in our therapeutic model: subepithelial collagen deposition was reduced in VacA-treated mice but not in mice treated with *H. pylori* lysate. Collagen formation around the airways is one hallmark of airway remodeling that plays a central role in airway obstruction for individuals with chronic asthma. This observation is therefore important for the potential therapeutic efficacy of VacA but needs to be investigated in more detail in follow-up projects.

To further assess the functionality of VacA, we repeated the experiments using a mutant form of VacA (VacA Δ6–27), which lacks vacuolating activity (30). Treatment with this mutant showed no suppressive effect on airway inflammation and goblet cell metaplasia, similar to previous findings in preventive models (18, 19). This suggests that a full-length functional form of the protein is required for the observed activity. To further evaluate the therapeutic potential of VacA, we compared purified VacA derived from *H. pylori* with a recombinantly generated form of VacA (rVacA). Both were found to be similarly effective in suppressing airway inflammation and goblet cell metaplasia.

Allergen-specific immunoglobulins were analyzed to further assess the immunomodulatory impact of VacA. While there did not appear to be any modulation of allergen-specific IgE and IgG1, a significant induction of HDM-specific IgG2b was detectable after administration of VacA, resulting in a decreased IgG1/IgG2b ratio. Interestingly, while allergen-specific IgG1 is associated with development of high affinity IgE responses (31, 32), induction of allergen-specific IgG2 responses are described as having the ability to antagonize IgE-mediated allergic reactions (33).

Attenuation of the disease phenotype accompanied by a IgG2b-biased shift of immunoglobulins has also been reported in models of allergic conjunctivitis testing the therapeutic effectiveness of either superoxide dismutase 3 (34) or rapamycin (35). Induction of IgG2b is mediated by Th1 cells (36), which have also been shown to counterbalance Th2-mediated pathology in asthma (37).

DCs in particular, but also macrophages, are mediators of *H. pylori*-driven immune suppression (18). The therapeutic administration of VacA resulted in DC with a slightly reduced MHCII expression in the draining LN; such “semi-mature” DCs are associated with T cell tolerance (38) and could therefore contribute to the reduced inflammatory phenotype seen with the acute therapeutic approaches. Murine *in vitro* and DC transfer approaches have previously highlighted the central role of DCs in *H. pylori*-mediated asthma suppression and showed that VacA contributed to the induction of Tregs (3). Altobelli et al. confirmed that VacA targets myeloid cells (DCs and macrophages) of the gastric lamina propria (18). In macrophages, VacA promotes the secretion of the anti-inflammatory molecules IL-10 and transforming growth factor-β, and thus probably promotes the development of Tregs locally and also in the periphery.

Also in our models, administration of VacA modulated Treg responses. In both the acute and secondary challenge models we found induction of Tregs locally at the application site (mesenteric LN). In the therapeutic model of allergic airway disease, increased proportions of Tregs were also detectable in the spleen and lung. These differences might occur to different treatment protocols and time points of analysis.

Initial analyzes of Treg subpopulations did not show any differences in their composition at the time of analysis. Proportion of CD3^+^/CD4^+^/FoxP3^+^/CD25^high^ cells which are considered as the dominant natural Treg population consisting of thymic derived and induced Tregs and CD3^+^/CD4^+^/FoxP3^+^/CD25^low^ cells which are described as ancillary regulatory arm with an higher activation threshold (23) were comparable. Moreover, also a detailed analyses of Neuropilin 1 and Helios expression in (Data not shown) in the population of FoxP3 positive Tregs in the therapeutic model did not reveal a VacA mediated induction of a specific Treg subtype like induced or naturally occurring Tregs (39).

Nevertheless, a VacA mediated induction of a particular Treg subpopulation is imaginable. It could be that at the time of the analysis differences between subpopulations are no longer visible due to migration movements of the cells within the body. Kinetic experiments, which are focus of ongoing work might help to reveal the impact of VacA on Treg subtypes.

In prophylactic neonatal studies, *H. pylori* infection has been reported to reduce susceptibility to allergic airway disease, an effect mediated by Tregs (40, 41). The inability of VacA-deficient mutants to induce Tregs *in vitro* and mediate asthma protection *in vivo*, and the observation that prophylactic application of purified VacA attenuated the development of allergic airway disease, underlines the capability of this protein to induce Treg-mediated immune suppression (9, 42). To analyze the role of Tregs in the present therapeutic model, depletion assays were performed using DEREG mice. In these animals, application of DT can temporally deplete Tregs, allowing direct assessment of the functional role of these cells. Interestingly, Treg depletion during VacA treatment completely abolished the beneficial effects of the molecule.

Although only modest effects on the number of Tregs and the activation status of DC were seen, the findings of the the depletion assay suggest that the suppressive effect of VacA is mediated *via* Tregs.

Using two different murine therapeutic models for allergic airway disease, the present study showed that *H. pylori*-derived VacA mediated immune suppression in adult animals and could therefore be useful as a therapeutic approach to asthma that might complement, or even replace, existing therapies.

To obtain initial data on whether VacA could be effective in humans, we compared its influence on the activation of DCs derived from humans (moDC) and mice (bone marrow derived DC). Application of VacA was associated with the upregulation of costimulatory molecules associated with the induction of adaptive immune responses, and was accompanied by the production of a variety of cytokines. In both mice and in human DC cultures, the immunosuppressive molecule IL-10 was detectable in increased concentrations. Induction of IL-10 is key for the protective effects mediated by various bacterial strains and compounds with well-documented therapeutic activity. In addition to extracts of *H. pylori* (9), other microbiota constituents such as *Bifidobacteria breve* (43) and *Faecalibacterium prausnitzii* (44) are also capable of inducing IL-10 secretion in DCs, and therefore the induction of beneficial Tregs. Interestingly, treatment with *F. prausnitzii* also induced tolerogenic factors in DCs (44). Moreover, VacA- and *F. prausnitzii*-treated naïve human moDCs showed induced expression of PD-L1, a surface molecule that is strongly associated with attenuation of T cell responses (45, 46). In addition to PD-L1, VacA is also capable of inducing production of ILT3, an inhibitory receptor that is expressed on tolerogenic DCs (47) and appears to be necessary for the induction of Tregs (48). Interestingly, VacA induces the expression of PD1 specifically in activated moDCs. Transfer models have shown that expression of PD1 on DCs attenuates the proliferation of antigen-specific CD8^+^ T cells (49). To assess Treg-inducing capacities in the current study, human moDCs were cultured with autologous T cells. VacA increased the proportion of Tregs under all conditions, underlining its regulatory properties in both murine and human systems.

Several epidemiological studies demonstrate that *H. pylori* strains containing particularly immunogenic forms of VacA are associated with increased gastric cancer risk. However, there is little direct evidence in animal models demonstrating that VacA contributes to the pathogenesis of gastric cancer (50). In the current study, no negative effects of treatment with VacA were seen in either the prophylactic or the therapeutic models nor in the murine and human *in vitro* models, suggesting that VacA may be a safe therapeutic intervention for the treatment of asthma.

However, the administration of such a therapeutic must be balanced to avoid potential unwanted side effects that could be mediated by the induction Tregs.

In summary, these data show suppression of allergic airway disease by administration of VacA in a therapeutic setting. VacA induced the generation of Tregs *via* modulation of DCs, and this induction of Tregs is a key event in VacA-mediated immune suppression in therapeutic asthma models.

Modulation of Treg responses might represent an interesting therapeutic approach not only for asthma but also for other diseases with an underlying exaggerated immune pathology. The prophylactic application of VacA demonstrated already beneficial effects in model of food allergy (19). Here but also in other allergic diseases is the therapeutic treatment approach of great interest and will be focus of future work.

VacA exerts similar tolerogenic effects on human moDCs, and can readily be produced in recombinant form, making it an attractive candidate for therapeutic intervention in patients with asthma.

## Data availability statement

The raw data supporting the conclusions of this article will be made available by the authors, without undue reservation.

## Ethics statement

The studies involving human participants were reviewed and approved by Ethics committee of the medical faculty of the University Duisburg-Essen, Germany (ethics vote 18-8069-BO). The patients/participants provided their written informed consent to participate in this study. The animal study was reviewed and approved by Landesamt für Natur, Umwelt und Verbraucherschutz North Rhine Westphalia, Germany; reference number: AZ 81-02.04.2018.A084.

## Author contributions

SR designed the experiments. SR, JR, HU, AC performed the experiments. SR and JR, analyzed the data. SR and CT conceived and supervised the project. EP, AW, GC, TC, AM supported the writing process and supplied VacA and animals. SR, JR and CT wrote the manuscript. All authors contributed to the article and approved the submitted version.
