# Supplementary material for: Treatment with Helicobacter pylori-derived VacA attenuates allergic airway disease
Source: Front Immunol. 2023 Jan 24;14:1092801. doi: 10.3389/fimmu.2023.1092801 (PMC9902502; doi:10.3389/fimmu.2023.1092801)
Supplement: Supplementary file 1 [file DataSheet_1.pdf]

Supplementary Material

Panel	murine Treg Analysis				
**Fluochrome**	**Specificity**	**Clone**	**Reactivity**	**Antibody Host Species**	**Manufacturer**
PerCP-Cy™5.5	CD3	145-2C11	mouse	rat	Biolegend
PE/Cyanine7	CD4	53-6.7	mouse	rat	Biolegend
Pe	CD25	PC61	mouse	rat	BD Pharmingens
Brilliant Violet 650™	CD25	PC61	mouse	rat	Biolegend
APC	FoxP3	FJK-16s	mouse	rat	Biolegend
**Panel**	**murine DC/B cell Analysis**				
**Fluochrome**	**Specificity**	**Clone**	**Reactivity**	**Antibody Host Species**	**Manufacturer**
FITC	MHCII	M5/114.15.2	mouse	rat	Biolegend
PE/Dazzle™ 594	CD19	6D5	mouse	rat	Biolegend
PE/Cyanine7	CD11c	N418	mouse	hamster	Biolegend
APC/Cyanine7	CD3	145-2C11	mouse	hamster	Biolegend
Alexa Fluor® 700	CD45	30F11	mouse	rat	Biolegend
Brilliant Violet 421™	Ly6c	HK1.4.	mouse	rat	Biolegend
Brilliant Violet 785™	B220	RA3-6B2	mouse/human	rat	Biolegend

Supplementary Table 1. Antibody List murine ex vivo Analysis

Panel	murine DC activation				
**Fluochrome**	**Specificity**	**Clone**	**Reactivity**	**Antibody Host Species**	**Manufacturer**
FITC	MHCII	M5/114.15.2	mouse	rat	Biolegend
PE	CD86	GL1	mouse	rat	Biolegend
PerCP-Cy™5.5	CD80	16-10A1	mouse	hamster	Biolegend
PE/Cyanine7	CD11c	N418	mouse	hamster	Biolegend
Pacific Blue	CD40	3/23	mouse	rat	Biolegend
NUV450	Zombie UV				Biolegend
**Panel**	**human DC activation**				
**Fluochrome**	**Specificity**	**Clone**	**Reactivity**	**Antibody Host Species**	**Manufacturer**
FITC	PD-L1	MIH3	human	mouse	Biolegend
PE/Dazzle™ 594	PD1	EH12.2.H1	human	mouse	Biolegend
PE/Cyanine7	ILT3	ZM4.1	human	mouse	Biolegend
Brilliant Violet 421™	HLA-DR	L243	human	mouse	Biolegend
Brilliant Violet 510™	CD80	2D10	human	mouse	Biolegend
Brilliant Violet 605™	CD40	5c3	human	mouse	Biolegend
Brilliant Violet 650™	CD11c	3.9	human	mouse	Biolegend
Brilliant Violet 785™	CD86	IT2.2	human	mouse	Biolegend
NUV450	Zombie UV				Biolegend
**Panel**	**human Treg Analysis**				
**Fluochrome**	**Specificity**	**Clone**	**Reactivity**	**Antibody Host Species**	**Manufacturer**
PercP-Cy5.5	CD4	RPAT4	human	mouse	Biolegend
APC	CD127	AO19D5	human	mouse	Biolegend
AF700	CD3	UCHT1	human	mouse	Biolegend
Brilliant Violet 421™	CD25	HIB 19	human	mouse	Biolegend
NUV450	Zombie UV				Biolegend

Supplementary Table 2. Antibody List murine/human in vitro Analysis

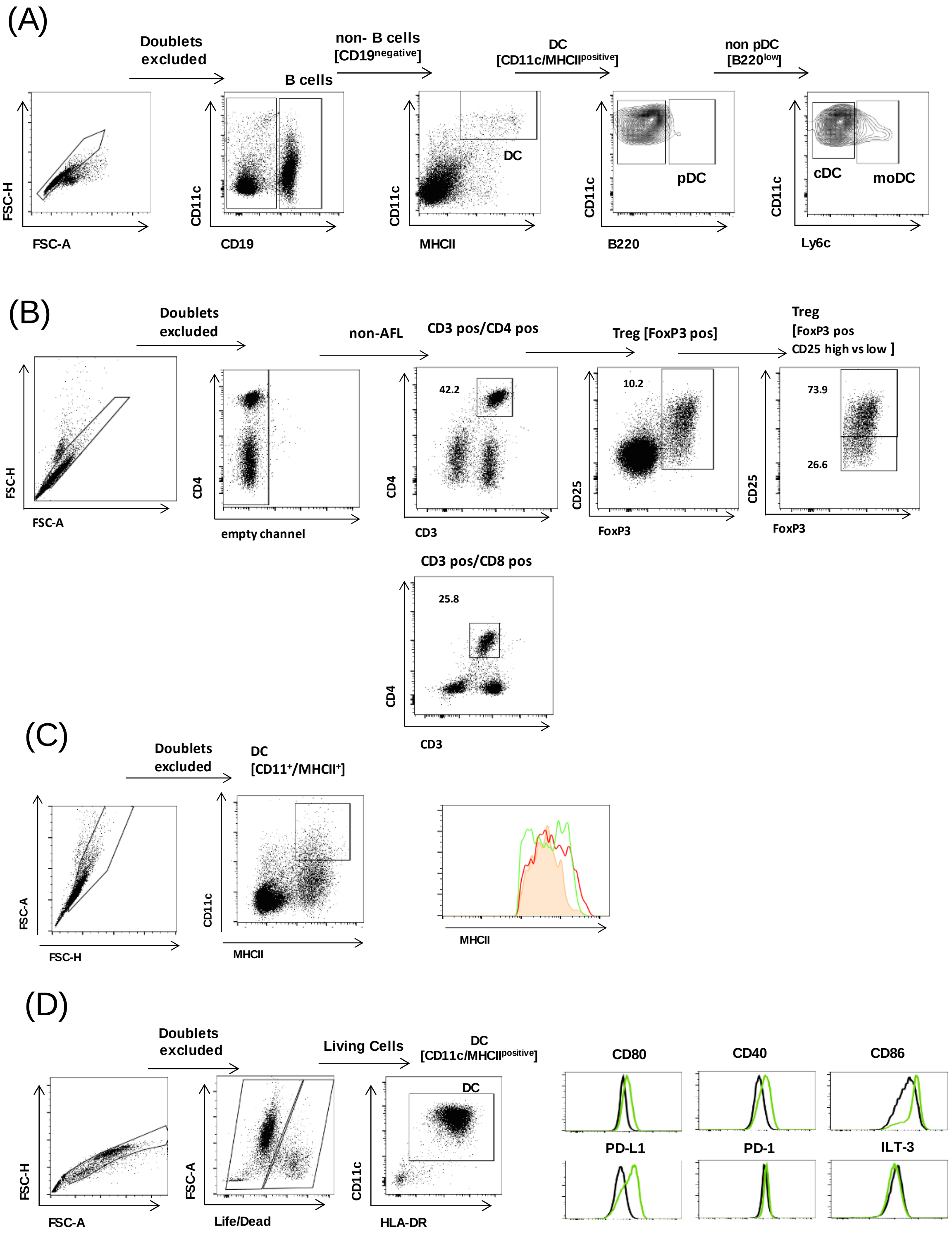

Supplementary Figure 1: Flow cytometry gating strategies B cells/DC Subtypes, Tregs and DC MHCII expression *ex vivo*/DC activation *in vitro*. A) The dot plots presented show the gating strategy used to analyze B cells and DC ex vivo. Following exclusion of doublets via FSC-A vs. FSC-H B cells were characterized as CD19 positive cells. Within the group of CD19 negative cells DC were analyzed as CD11c/MHCII positive cells. To further distinguish subpopulation expression

of B220 was analyzed and positive cells were characterized as pDC. Further, Ly6c expression was analyzed on B220 negative cells. Ly6c negative cells were identified as cDC and positive cells as moDC. B) The gating strategy shows the in vitro analysis of regulatory T cells ex vivo. First, debris and doublets were excluded based on size and shifting properties seen by analysis of FSC-Height vs. FSC-Area. Autofluorescent cells, positive cells in an empty channel were excluded. Based on the expression of CD8/CD3 cytotoxic T cells were differentiated from CD3/CD4 positive T Helper cells. Tregs were then defined as FoxP3-positive cells within the population of CD3/CD4 positive cells. Treg subpopulations were further subdivided in $CD25^+$ cell and $CD25^-$ cells. C) The gating demonstrates identification of DC and analysis of their MHCII expression in tLN: First, debris and doublets were excluded based on size and shifting properties seen by analysis of FSC-Height vs. FSC-Area. DC were identified by the expression of CD11c and MHCII. Within the population mean fluorescence intensity of MHCII was analyzed. Histogram shows MHCII expression in HDM/PBS (shaded orange), HDM/HDM (red line) and HDM/HDM VacA animals (green line). D) The gating strategy shows the in vitro analysis of DC activation using human DC as an example. Following exclusion of doublets living cells were identified based on the reduced expression of a life/dead marker. Within the living cells DC were characterized as CD11c/HLA-DR positive cells. Within the DC population surface expression of the markers shown in the histograms was analyzed. Histograms show the expression of the indicated markers on naïve DC (black line) and naïve DC treated with VacA (green line).

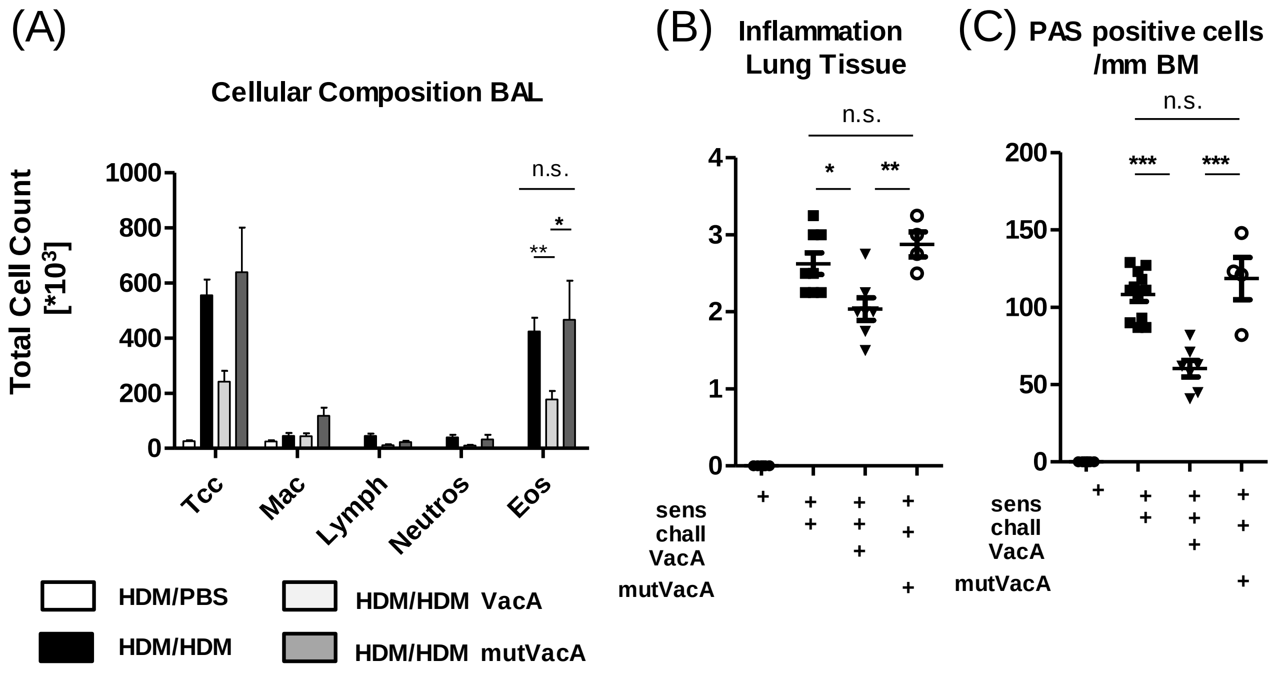

Supplementary Figure 2: Effect of a VacA mutant on asthma hallmarks in an acute murine asthma model. A) **Cellular composition of bronchoalveolar lavage (BAL) fluid**: The positive control (HDM/HDM, black bars) showed increased total cell count (Tcc), macrophages (Mac), lymphocytes (Lymph), neutrophils (Neutros) and eosinophils (Eos) compared with the negative Control (HDM/PBS, white bars). Eosinophil numbers were reduced in animals treated with VacA (HDM/HDM VacA, light gray) and comparable to the positive control in animals treated with the VacA mutant (HDM/HDM mutVacA, dark gray). **B**: **Inflammation in lung tissue:** Scatter plot shows inflammation score for HDM/PBS, HDM/HDM, HDM/HDM VacA or HDM/HDM mutVacA animals. **C**: **Mucus-producing cells in lung airways:** Scatter plot shows averaged number of mucus-producing cells/mm basal membrane in HDM/PBS, HDM/HDM and HDM/HDM VacA or HDM/HDM mutVacA animals.

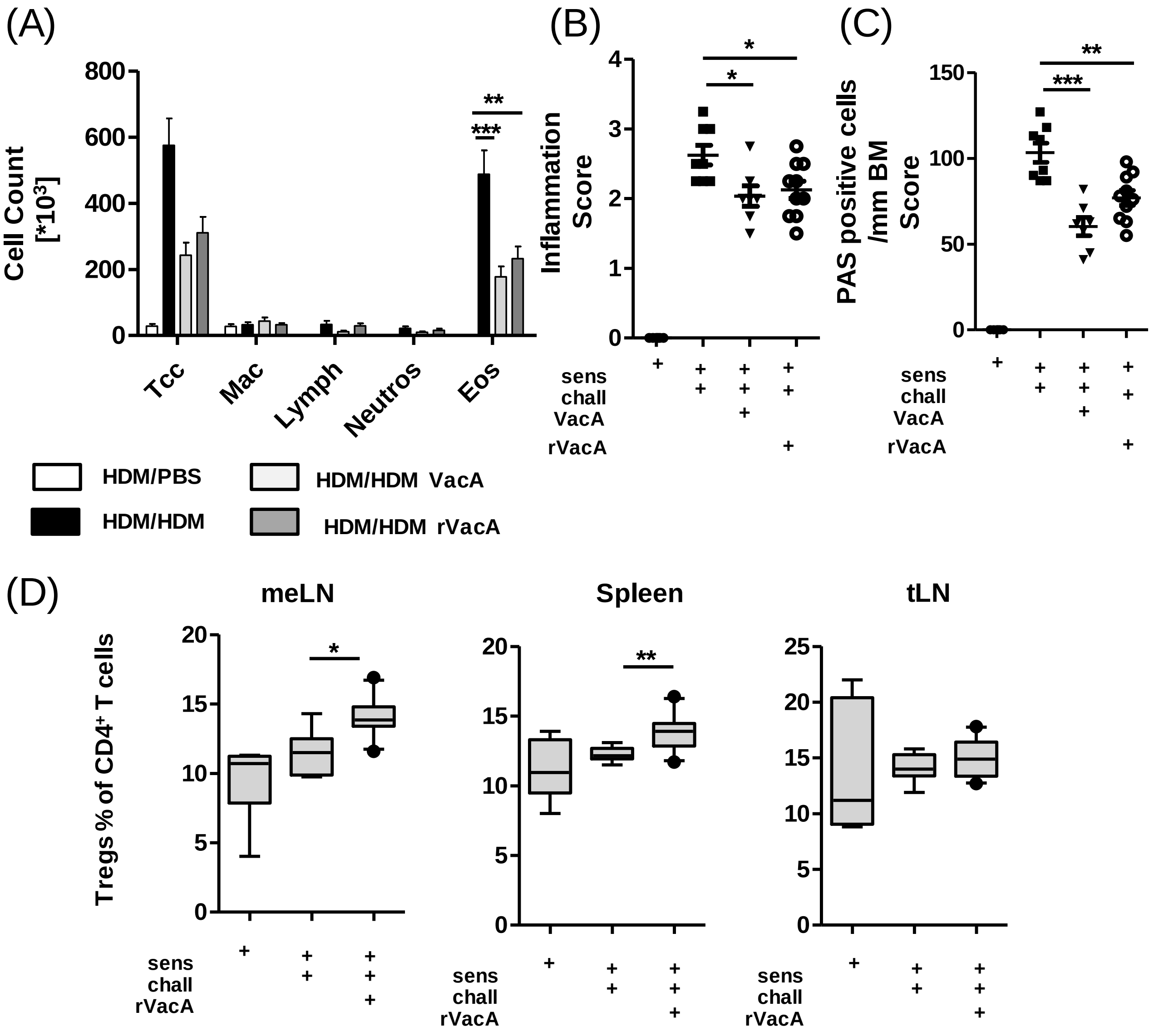

Supplementary Figure 3: Recombinant produced VacA attenuates allergic airway disease in adult mice. A) Cellular composition of bronchoalveolar lavage (BAL) fluid: The positive control (HDM/HDM, black bars) showed increased total cell count (Tcc), macrophages (Mac), lymphocytes (Lymph), neutrophils (Neutros) and eosinophils (Eos) compared with the negative Control (HDM/PBS, white bars). Numbers eosinophils were reduced detectable in animals treated with VacA (HDM/HDM VacA, light gray) or rVacA (HDM/HDM VacA, dark gray). **B**) **Inflammation in lung tissue:** Scatter plot shows inflammation scores for HDM/PBS, HDM/HDM, HDM/HDM VacA or HDM/HDM rVacA animals. **C**) **Mucus-producing cells in lung airways:** Scatter plot shows averaged number of mucus producing cells/mm basal membrane in HDM/PBS, HDM/HDM and HDM/HDM VacA or HDM/HDM rVacA animals. **D**) Box plots (Whiskers 10-90 percentile) show proportion of CD25/FoxP3 positive cells within the CD4/CD3 positive T helper cell population in

mesenteric lymph nodes (meLN), spleen and lung draining lymph nodes (tLN) of negative and positive controls, and rVacA-treated animals. **B-D**: Each point represents one animal, Data are results from two independent experiments, n=6–10 per group; Analysis of variance, $*p<0.05$, $**p<0.01$, $***p<0.001$.

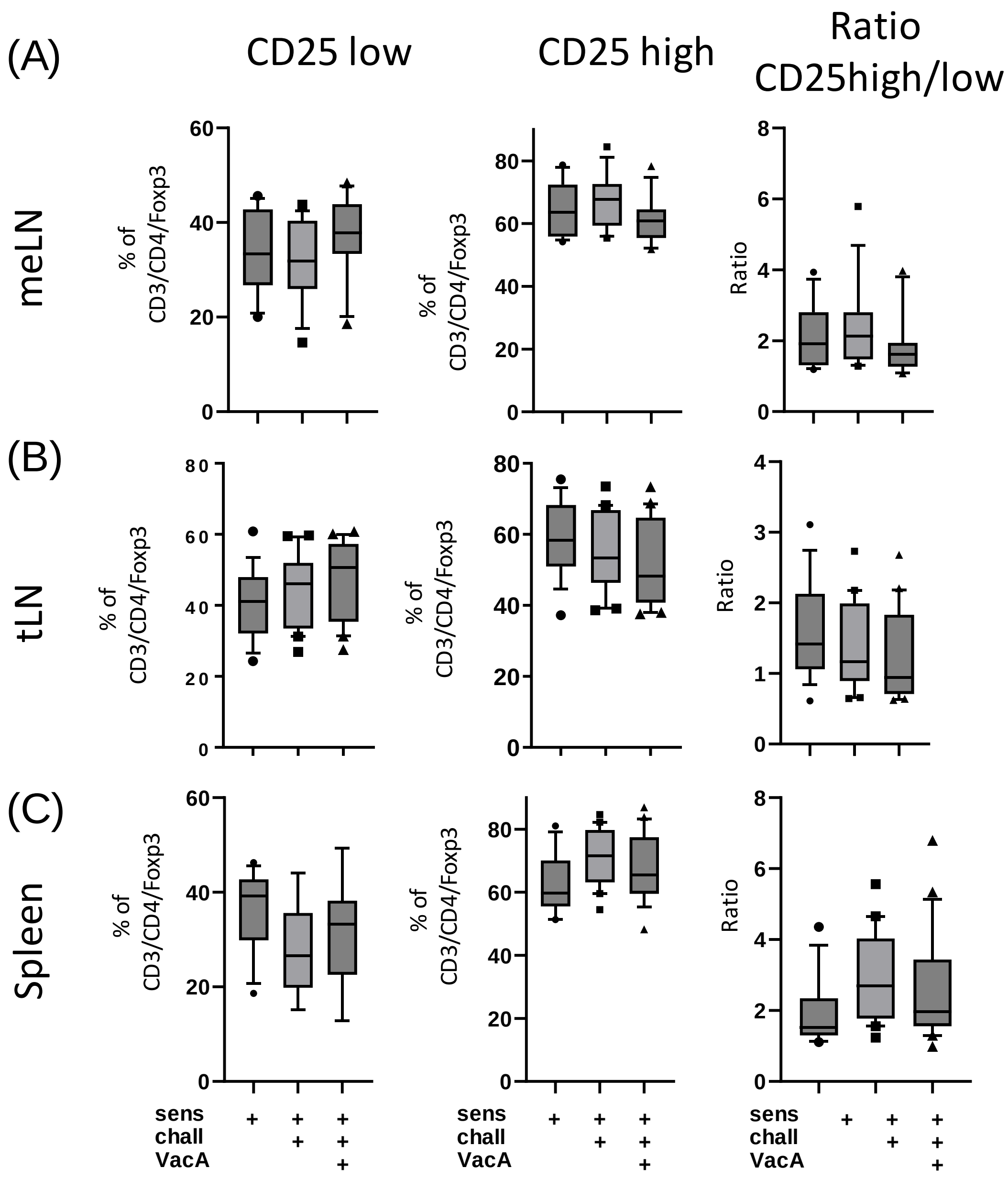

Supplementary Figure 4: Treatment with VacA did not affect Treg Subtypes Within the population of CD3$^+$CD4$^+$/FoxP3$^+$ cells CD25^{low} and CD25^{high} cells were analyzed. Boxplots (Whiskers 10-90 percentile) show percentage of CD25^{low}, CD25^{high} and Ratio of CD25^{high}/CD25^{low} Tregs in A) meLN B) tLN and C) spleen of HDM/PBS, HDM/HDM and HDM/HDM VacA-treated mice. Results are from 4-5 independent experiments, n=13-21 mice per group.

Analysis of variance, *p<0.05, **p<0.01, ***p<0.001.

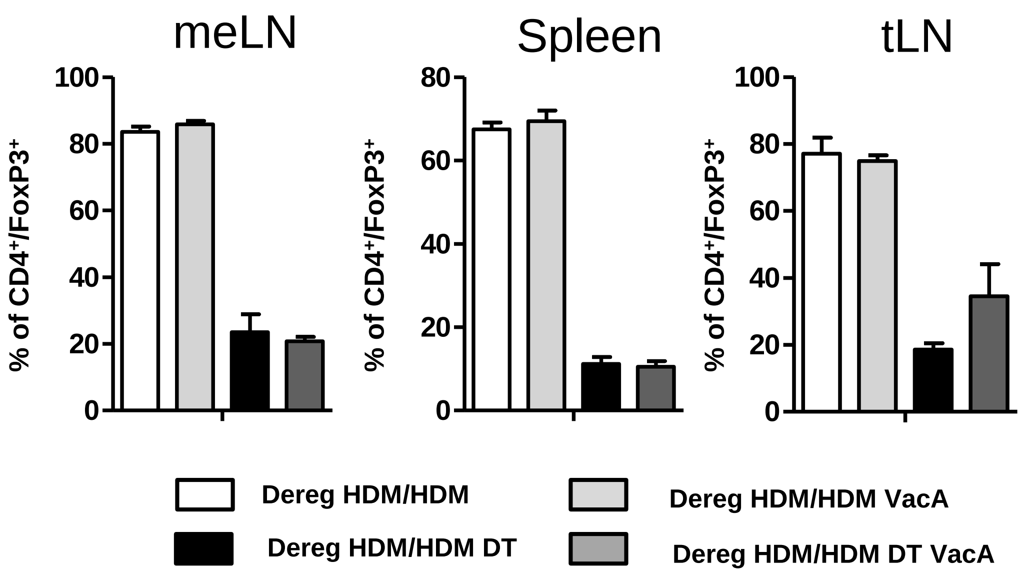

Supplementary Figure 5:

Bar graphs show the proportion (%) of green fluorescent protein (GFP)-positive regulatory T cells in DEREG mice sens/chall (white bars), DEREG mice sens/chall treated with VacA (light gray bars), DEREG mice sens/chall with DT depletion (black bars) and DEREG mice sens/chall treated with VacA and DT depletion (dark gray bars).

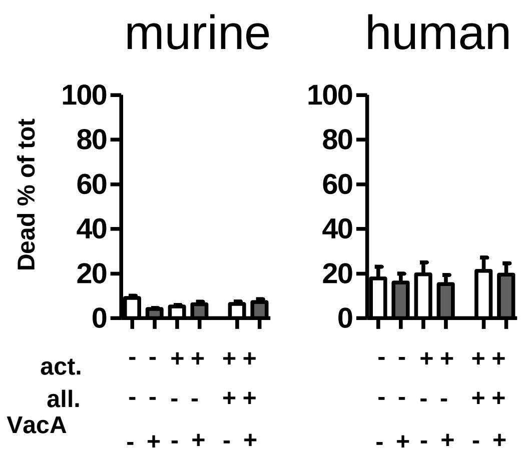

Supplementary Figure 6: VacA did not demonstrate cytotoxic effects. Bar graphs demonstrate % of dead cells in the in vitro cultures of murine and human naïve, activated and activated VacA supplemented dendritic cells.
